# Perfectly Wetting Mixtures of Surfactants from Renewable Resources: The Interaction and Synergistic Effects on Adsorption and Micellization

**DOI:** 10.1007/s11743-016-1793-z

**Published:** 2016-02-13

**Authors:** Patrycja Szumała, Alicja Mówińska

**Affiliations:** Department of Fats and Detergents Technology, Faculty of Chemistry, Gdansk University of Technology, Narutowicza 11/12, 80-233 Gdansk, Poland

**Keywords:** Surface property, Wetting, Contact angle, Synergy, Adsorption

## Abstract

This paper presents a study of the surface properties of mixtures of surfactants originating from renewable sources, i.e., alkylpolyglucoside (APG), ethoxylated fatty alcohol (AE), and sodium soap (Na soap). The main objective was to optimize the surfactant ratio which produces the highest wetting properties during the analysis of the solution of the individual surfactants, two- and three-component mixtures, and at different pH values. The results showed the existence of a synergistic effect in lowering the interfacial tension, critical micelle concentration and the formation of mixed micelles in selected solutions. We found that best wetting properties were measured for the binary AE:APG mixtures. It has been demonstrated that slightly lower contact angles values were observed on Teflon and glass surfaces for the AE:APG:soap mixtures but the results were obtained for higher concentration of the components. In addition, all studied solutions have very good surface properties in acidic, basic and neural media. However, the AE:soap (molar ratio of 1:2), AE:APG (2:1) and AE:APG:soap (1:1:1) compositions improved their wetting power at pH 7 on the aluminium and glass surfaces, as compared to solutions at other pH values tested (selected Θ values close to zero—perfectly wetting liquids). All described effects detected would allow less surfactant to be used to achieve the maximum capacity of washing, wetting or solubilizing while minimizing costs and demonstrating environmental care.

## Introduction

Industrial detergents, wetting agents and conditioning compositions are generally mixtures of different types of surfactants. Those mixtures often exhibit a favorable synergistic effect, defined as an improvement in a property compared to that attained by either of the pure surfactants, which allows the use of smaller amounts of components while maintaining the desired properties [1]. As a result, a reduction in the production costs and a positive impact on the environment can be achieved.

Surfactants such as alkylpolyglucosides (APG) and ethoxylated fatty alcohols (AE) are widely used because they are not only readily biodegradable and highly efficient on fatty soils [2] but also come from renewable sources. APG are nonionic compounds with excellent ecological, toxicological properties [3] and interfacial properties (low interfacial tension, and hydrotropic properties) [4]. They are used in cleaning and as detergent products because of their good foaming properties and skin compatibility [5]. AE are the most common group of nonionic surfactants. Their main application is in washing powders and liquids, cleaning products, cosmetics and they are classified as easily biodegradable compounds [6]. Therefore studies on surface activity of such surfactants mixtures may be helpful in determining their applicability in commercial products or even contribute to the development of technical specifications for future applications in many different industries that use surfactants.

One of the most important surfactant surface properties is wettability which is described in a number of scientific reports. Jurado et al. [2, 7], in analyzing the wetting power and the detergency of solutions of APG and AE, assumed that surfactant mixtures most efficient at cleaning also show the highest wetting power. In a different work [8], it was observed that the wetting ability of an aqueous mixture containing APG can be improved by including AE.

However, depending on the pH, the surface/material and the concentration of components wetting power can vary. The surface properties are also associated with the interaction between surfactants and their synergistic action. In the present work, not only the wettability but also the synergistic effect on adsorption and micellization of different aqueous mixtures of APG, AE and sodium soap (Na soap) were studied in order to identify mixtures with the highest surface activity. From our knowledge, no studies have been published on surface behavior of these very often used surfactants in their mixtures. Sodium soap is one of the best known surfactants, which is also obtained from the raw materials. The influence of the surfactants concentration, molar ratio, and the pH values of the mixtures on the surface tension, the contact angle and intermolecular interactions have been analyzed.

## Experimental

### Materials

Alkylpolyglucoside (APG, mixture of alkyl chain C_8_–C_10_, degree of polymerization = 1.6, average molecular formula C_8.6_G_1.6_ [9] Glucopon 215 UP) were purchased from Brenntag (UK and Ireland), sodium soap (Na soap, composition of C_12_–C_18_, average molecular formula C_13.4_Na, Prisavon 1873) was obtained from Coda (UK), and ethoxylated (7EO) lauryl alcohol C_12_–C_14_ (AE, average molecular formula C_13_E_7_, Rokanol L7) was purchased from PCC Exsol (Poland). The water used for the preparation of the solution was double-distilled with a surface tension of 73.1 ± 0.5 mN/m at 20 °C. The solutions at suitable pH were made with Britton–Robinson buffer [10] and the required chemicals, i.e., phosphoric acid, acetic acid, boric acid and sodium hydroxide were purchased from Sigma Aldrich (Poland). The pH of each prepared solution was tested with a pH meter (Elmetron, Poland).

### Methods

The air–water surface tensions were measured using a pendant drop method (Krüss Drop shape analyzer DSA 10, Hamburg, Germany). Measurements were performed by producing a pendant drop (top-to-bottom) of aqueous solutions, recording and analyzing the drop shape with a CCD. After drop equilibration, three results of surface tension were measured. For each test solution three drops were analyzed. The final result is the arithmetic average of nine measurements.

Contact angles were studied using the same DSA10 (Krüss). The kinetics of spreading was investigated on three different surfaces: aluminium, glass and Teflon. The solid surfaces were cleaned with acetone and blown through with compressed air. After solvent evaporation the measurement of the contact angle for the standard liquid, i.e., double distilled water, was made in order to confirm the purity of the surface. Measurements of the contact angle were made 30, 40 and 50 s after drops had settled on the tested surface. For each solution, five drops were analyzed. The final result is the arithmetic average of fifteen measurements. The model for the drop shape analysis depends on the sizes of the drop and the contact angle and the symmetry and shape of the drop. In our study, we mostly chose the Young–Laplace fit (sessile drop fitting, suitable for symmetrical drop shapes) and circle fitting method (height–width method).

In order to determine the surface activity of the single/mixed surfactants, a number of parameters were calculated:

1. The surface pressure (П_CMC_), defined as the difference between the surface tension of the solvent (*γ*_0_) and the solution (*γ*_CMC_), Eq. 1 [11].
  1 $$\varPi_{\text{CMC}} = \gamma_{0} - \gamma_{\text{CMC}}$$
2. The maximum surface excess concentration, (*Γ*_max_), an adsorption parameter designated by a graphic method using Eq. 2 [12, 13].
  2 $$\varGamma_{\hbox{max} } = - \frac{a}{RTn}$$
  where *a* is the slope of the curve $\gamma = f \, \left( {\ln \, c} \right)$ before CMC (N/m), *R* is the gas constant [8.314 (J/mol K)], *T* is the temperature (K), *n* is the number of individuals that may be present at the interface.
3. The minimum areas per molecule, *A*_min_, from the Eq. 3 [13].
  3 $$A_{\hbox{min} } = \frac{{10^{18} }}{{N_{\text{av}} \cdot \varGamma_{\hbox{max} } }}$$
  where *N*_av_ is Avogadro’s number [6.022 × 10^23^ (1/mol)].
4. The efficiency parameter in the interfacial tension reduction, *pC*_20_, from Eq. 4 [14].
  4 $$pC_{20} = - \log \, \left( {C_{20} } \right)$$
  where *C*_20_ is the molar surfactant concentration needed to produce a 20 mN/m reduction in the interfacial tension.

## Results and Discussion

### Surface Activity of Single Surfactant Solutions

**Fig. 1:**
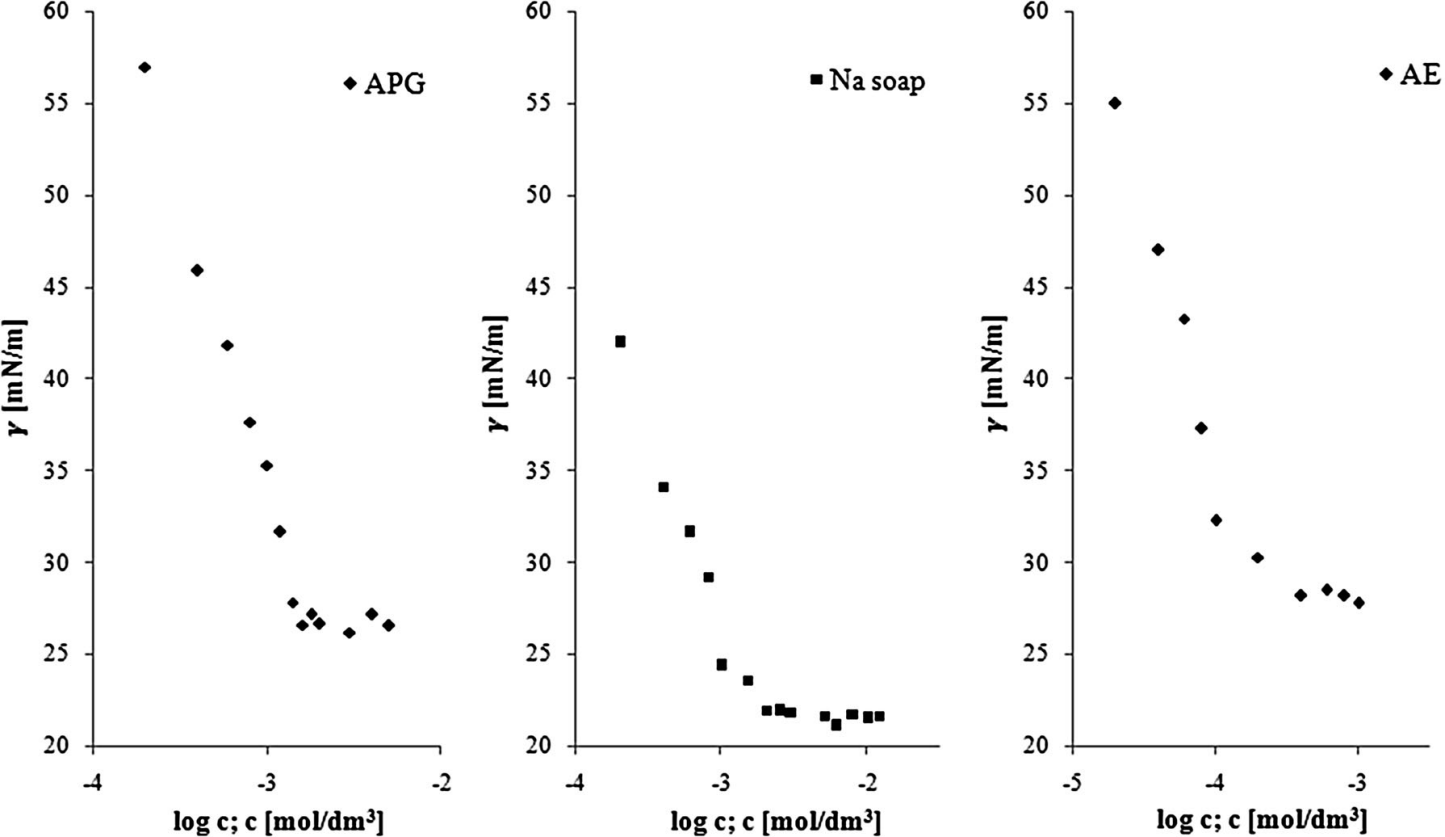
Surface tension *vs* surfactants concentration for APG, Na soap and AE

In order to determine the surface activity of individual surfactants, i.e., AE, APG and soaps, measurements of the surface tension were made (Fig. 1). From each graph the value of the critical micelle concentration (CMC) and surface tension at CMC (*γ*_CMC_) were determined. Tables 1 and 2 show the results of the calculated parameters of micellization and adsorption. The lowest value of CMC (i.e., 2.6 × 10^−4^ mol/dm^3^) was found for AE. Literature data [15] gives even lower CMC for C_12_EO_7_, but this might be due to the different average carbon chain length and a different distribution of the quantity of EO groups in the compound analyzed in that study. The other surface properties are in agreement with the literature values [15, 16]. AE also had the highest efficiency in surface tension reduction. The high *pC*_20_ value indicates that surfactant concentration is close to the minimum concentration required to produce maximum adsorption at the interface [17].

**Table 1:** The parameters of micellization of individual surfactant solutions

Surfactant	CMC × 10^3^ (mol/dm^3^)	*γ*_CMC_ (mN/m)	П_CMC_ (mN/m)
APG	1.63 ± 0.06	26.9 ± 0.4	46.3
Na soap	1.75 ± 0.05	21.9 ± 0.4	51.3
AE	0.26 ± 0.02	28.6 ± 0.3	44.5

**Table 2:** The adsorption parameters of individual surfactant solutions

Surfactant	*Γ* × 10^6^ (mol/m^2^)	*A*_min_ (nm^2^)	*C*_20_ × 10^4^ (mol/dm^3^)	*pC*_20_	CMC/*C*_20_
APG	5.79 ± 0.10	0.29 ± 0.005	2.6	3.6	6.3
Na soap	1.78 ± 0.04	0.93 ± 0.020	0.5	4.3	34.3
AE	3.79 ± 0.03	0.44 ± 0.003	0.2	4.7	13.6

On the other hand, Na soap had the highest CMC/*C*_20_ ratio. This parameter is a measure of the tendency of the surfactant to adsorb at the air/solution interface relative to its tendency to form micelles [18]. Thus Na soap had also great efficiency of adsorption (*pC*_20_ value). The interfacial pressure *П*_max_ attained by Na soap had the highest value (Table 1), and this parameter may be treated as a measure of the effectiveness of the interfacial tension reduction [17].

APG had the largest value of the maximum surface excess *Γ*_max_, and the smallest value of area occupied by one molecule (Table 2). This means that the adsorbed monolayer formed by this surfactant is most closely packed. It is common that surfactants with straight chains and large head groups (relative to the tail cross section) favor close, effective packing at the interface [17]. The value of adsorption and micellization parameters are in agreement with the literature values [18].

As can be seen all three surfactants have very good but different surface properties. However, most industrial products that contain surfactants are mixed solutions of surface active agents. Therefore, we analyzed compositions of these three compounds in the different molar ratios.

### The Surface Activity and Interaction of Binary and Ternary Mixtures of Surfactants

**Fig. 2:**
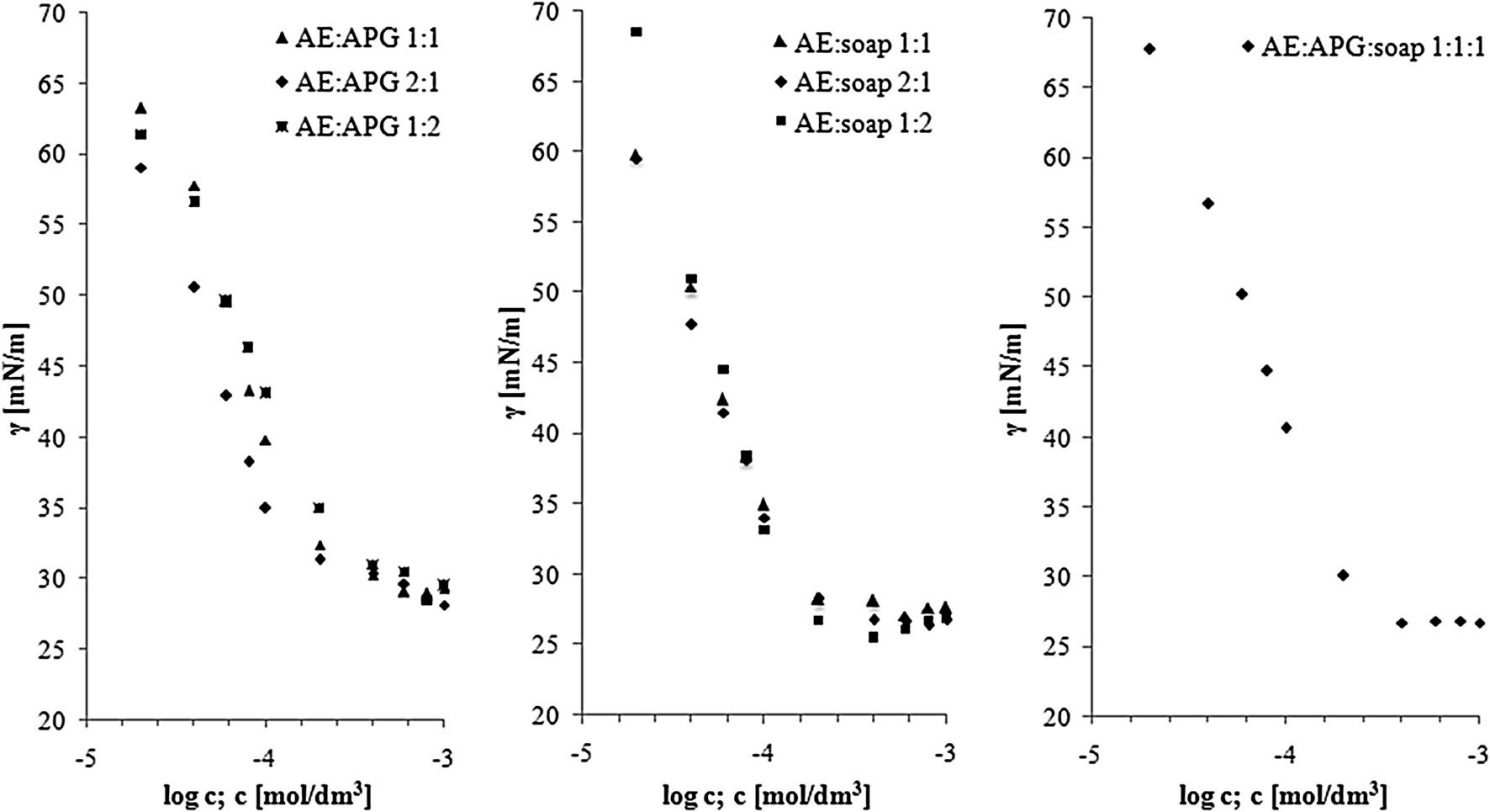
Surface tension *vs* surfactants concentration for mixed surfactant solutions: AE:APG, AE:Na soap and AE:APG:Na soap respectively, at different molar ratios

In order to investigate the efficiency of surface tension reduction and CMC values, the following mixtures of surfactants were prepared and analyzed: (a) AE:soap, molar ratio 1:1, 1:2, 2:1; (b) AE:APG, molar ratio 1:1, 1:2, 2:1; (c) AE:APG:soap, molar ratio 1:1:1. Figure 2 shows the results of surface tension as a function of concentrations of surfactants. It has been found that solution of AE:soap 1:2 has the lowest value of *γ*_CMC_ (~26 mN/m). This result indicates the maximum capacity of this mixture for lowering the surface tension. With the decrease in AE/soap ratio (2:1, 1:1. 1:2 respectively) we observed decreased CMC and *A*_min_ values were found, compared to the other binary solutions with soap (Table 3). On the other hand, the mixture of AE:soap in the ratio of 2:1 had the highest efficiency in reducing the surface tension (*C*_20_ = 2.8 × 10^−5^ mol/dm^3^). Our calculations suggest that the lowest value of CMC can by observed for the mixture of AE:APG (molar ratio 2:1, CMC = 1.18 × 10^−4^ mol/dm^3^). This mixture showed highest maximum surface excess and lowest *A*_min_ (Table 4). While the three-component solution had a relatively high CMC value, it significantly reduces the surface tension of water (*γ*_CMC_ = 26.76 mN/m).

**Table 3:** The parameters of micellization of surfactant mixture solutions

Surfactant mixture	Molar ratio	CMC × 10^4^ (mol/dm^3^)	*γ*_CMC_ (mN/m)	П_CMC_ (mN/m)
AE:Na soap	1:1	1.52 ± 0.01	28.3 ± 0.4	44.8
	2:1	1.90 ± 0.06	26.7 ± 0.1	46.4
	1:2	1.39 ± 0.01	26.1 ± 0.2	47.1
AE:APG	1:1	2.13 ± 0.02	30.7 ± 0.4	42.5
	2:1	1.18 ± 0.01	32.6 ± 0.3	40.5
	1:2	2.69 ± 0.16	31.8 ± 0.6	41.4
AE:APG:Na soap	1:1:1	2.41 ± 0.05	26.8 ± 0.2	46.4

**Table 4:** The adsorption parameters of surfactant mixture solutions

Surfactant mixture	Molar ratio	*Γ*_max_ × 10^6^ (mol/m^2^)	*A*_min_ (nm^2^)	*C*_20_ × 10^4^ (mol/dm^3^)	*pC*_20_
AE:Na soap	1:1	4.23 ± 0.01	0.39 ± 0.001	0.3	4.5
	2:1	4.18 ± 0.01	0.40 ± 0.001	0.3	4.6
	1:2	5.19 ± 0.04	0.32 ± 0 002	0.4	4.4
AE:APG	1:1	5.79 ± 0.06	0.29 ± 0.003	0.5	4.4
	2:1	6.16 ± 0.03	0.27 ± 0.001	0.3	4.5
	1:2	4.80 ± 0.02	0.35 ± 0.001	0.5	4.4
AE:APG:Na soap	1:1:1	5.00 ± 0.01	0.33 ± 0 001	0.5	4.3

Also important is the fact that all surfactant compositions, except AE:APG 1:2, exhibited lower CMC values than solutions of the individual components. This suggests a synergistic effect in lowering the CMC.

Interaction of the surfactant molecules in mixtures can result in an improvement or deterioration of their properties [16]. In order to determine the nature of the interactions, the following parameters were calculated:

1. The parameters of intermolecular interactions for surfactant mixtures in the mixed monolayer, $\beta_{LL}^{\sigma }$ (Eqs. 5, 6):
  5 $$\beta_{LL}^{\sigma } = \frac{{\ln \left( {\frac{{C_{1} }}{{C_{1}^{0} \cdot X_{1} }}} \right)}}{{\left( {1 - X_{1} } \right)^{2} }}$$
  6 $$\frac{{X_{1}^{2} \cdot \ln \left( {\frac{{C_{1} }}{{C_{1}^{0} \cdot X_{1} }}} \right)}}{{\left( {1 - X_{1} } \right)^{2} \cdot \ln \left( {\frac{{C_{2} }}{{C_{2}^{0} \cdot \left( {1 - X_{1} } \right)}}} \right)}} = 1$$
  where *X*_1_, the mole fraction of surfactant 1 in the total mixed surfactants monolayer, $C_{1}^{0}$, $C_{2}^{0}$, the solution phase molar concentrations of surfactant 1, surfactant 2 in the systems contained one surfactant, *C*_1_, *C*_2_, the solution phase molar concentrations of surfactant 1, surfactant 2 in their mixture, respectively, required to produce a given interfacial tension value, $\beta_{LL}^{\sigma }$, the molecular interaction parameter for mixed monolayer formation at the aqueous solution interface.
2. The parameter of intermolecular interactions between the surfactants in the mixed micelle, $\beta_{LL}^{M}$, Eqs. 7, 8 [18]:
  7 $$\frac{{\left( {X_{1}^{M} } \right)^{2} \cdot \ln \left( {\frac{{{\text{CMC}}_{1} }}{{{\text{CMC}}_{1}^{0} \cdot X_{1}^{M} }}} \right)}}{{\left( {1 - X_{1}^{M} } \right)^{2} \cdot \ln \left( {\frac{{{\text{CMC}}_{2} }}{{{\text{CMC}}_{2}^{0} \cdot \left( {1 - X_{1}^{M} } \right)}}} \right)}} = 1$$
  8 $$\beta_{LL}^{M} = \frac{{\ln \left( {\frac{{{\text{CMC}}_{1} }}{{{\text{CMC}}_{1}^{0} \cdot X_{1}^{M} }}} \right)}}{{\left( {1 - X_{1}^{M} } \right)^{2} }}$$
  where $X_{M}^{1}$, the molar fraction of surfactant 1 (AE) in a mixture, in the interfacial area, after reaching CMC, ${\text{CMC}}_{1}^{0}$, critical micelle concentration of surfactant 1 (mol/dm^3^), CMC_2_, critical micelle concentration of surfactant 2 in a mixture (mol/dm^3^), ${\text{CMC}}_{2}^{0}$, critical micelle concentration of surfactant 2 (mol/dm3).
  The negative value of β_LL_ parameter indicates attractive interactions between the surfactant molecules adsorbed at the interface or in the bulk solution, and the positive value indicates a repulsive interaction [19].

On the basis of the individual parameter values, it can also be determined:

1. Existence of synergism in the efficiency of reducing the interfacial tension [19, 20], if the following relations are true:
  9 $$\beta_{{_{LL} }}^{{^{\sigma } }} < 0;$$
  10 $$\beta_{LL}^{\sigma } \left| > \right|\ln \left( {C_{1}^{0} /C_{2}^{0} } \right)|$$
2. The synergism in mixed micelle formation occurs when the CMC of the surfactant mixture has a lower value than the CMC of individual surfactants forming micelles. The fulfillment of the following conditions indicates synergism in mixed micelle formation [20]:
  11 $$\beta_{{_{LL} }}^{{^{M} }} < 0;$$
  12 $$\beta_{LL}^{\sigma } \left| > \right|\ln ({\text{CMC}}_{1}^{0} /{\text{CMC}}_{2}^{0} )|;$$
3. Synergy in the effectiveness of the interfacial tension reduction occurs when γ_CMC_ of the surfactant mixture is lower than the interfacial tension for the individual surfactants [20]. To obtain synergy, the following conditions must be met:
  13 $$\beta_{LL}^{\sigma } - \beta_{LL}^{M} < \, 0;$$
  14 $$|\beta_{LL}^{\sigma } - \beta_{LL}^{M} \left| { \, > \, } \right|(\gamma^{0} {\text{CMC}}_{1} - \gamma^{0} {\text{CMC}}_{2} )/a|$$
  where *a* is the slope of the curve $\gamma = \, f \, \left( {\ln \, c} \right)$ for the solution of a single surfactant which is characterized by a higher value of the interfacial tension at the CMC.

Table 5 shows the results of the measurement of intermolecular interactions in solutions. In all surfactants mixtures, the $\beta_{LL}^{\sigma }$ coefficient values are negative. For all two-component systems the dependency of $\beta_{LL}^{\sigma } \left| > \right|\ln \left( {C_{1}^{0} /C_{2}^{0} } \right)|$ was true, which means that in all solutions there is a synergism in lowering the interfacial tension. The $\beta_{LL}^{M}$ values were found negative, and in all the mixtures the relations $\left| {\beta_{LL}^{\sigma } - \beta_{LL}^{M} } \right| > \left| {(\gamma^{0} {\text{CMC}}_{1} - \gamma^{0} {\text{CMC}}_{2} )/a} \right|$ existed. This implies that synergism in mixed micelles formation in all systems was observed.

**Table 5:** The values of the intermolecular interactions in solutions of surfactant mixtures

Surfactants mixture	Molar ratio	X_1_	$\beta_{LL}^{\sigma }$	$\ln \left( {C_{1}^{0} /C_{2}^{0} } \right)$	$X_{1}^{M}$	$\beta_{LL}^{M}$	$\ln \left( {{\text{CMC}}_{1}^{0} /{\text{CMC}}_{2}^{0} } \right)$
AE:Na soap	1:1	0.6	−4.1	−1.2	0.6	−5.5	−1.9
	2:1	0.7	−3.5	−1.2	0.7	−4.2	−1.9
	1:2	0.5	−5.1	−1.2	0.6	−6.3	−1.9
AE:APG	1:1	0.7	−2.5	−1.9	0.7	−3.9	−1.9
	2:1	0.7	−4.3	−1.9	0.7	−6.3	−1.9
	1:2	0.7	−2.0	−1.9	0.6	−3.5	−1.9

### Wetting Properties of the Surfactant Mixtures

**Fig. 3:**
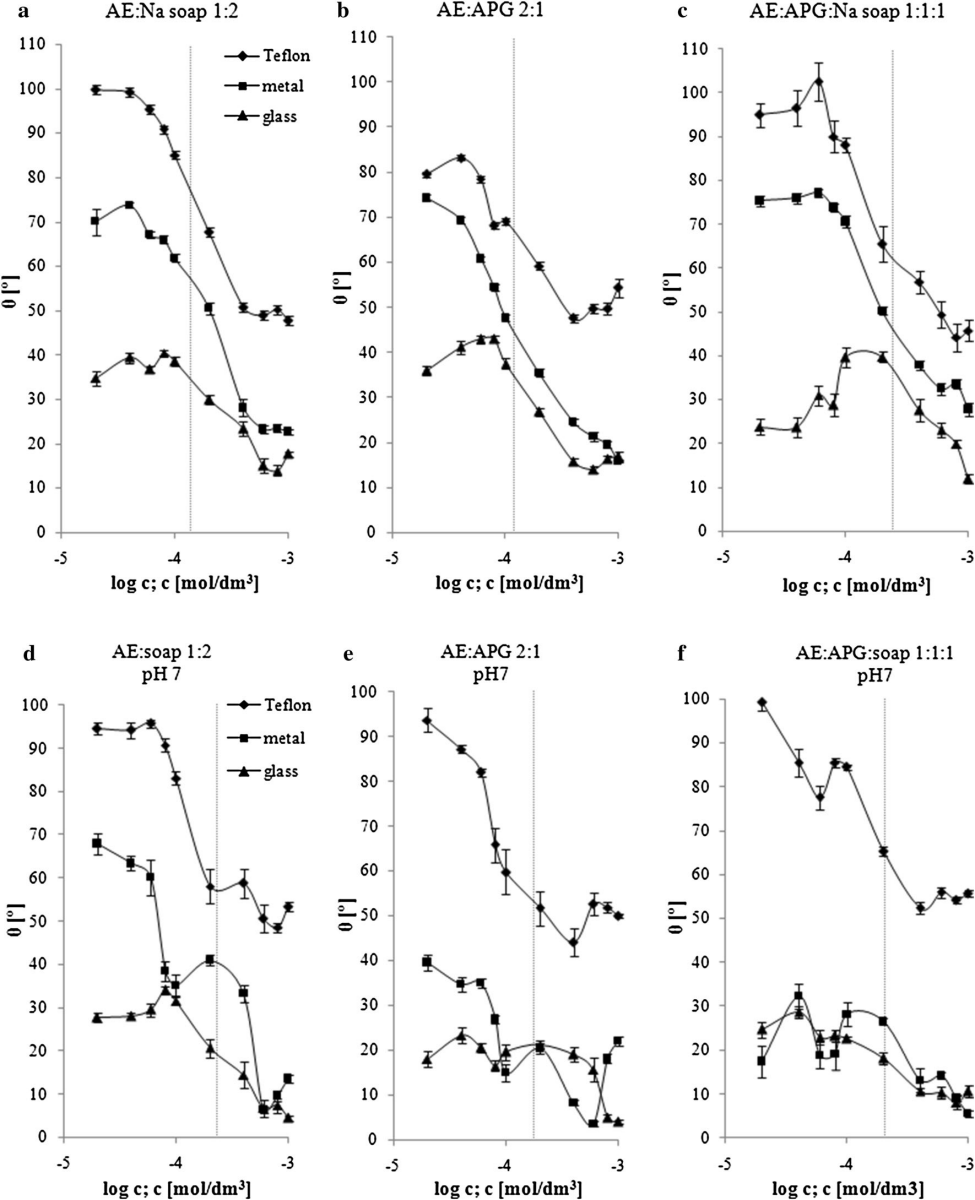
A plot of the contact angle with the concentration of the surfactant mixtures at different molar ratios: AE:Na soap (1:2), AE:APG (2:1), AE:APG:Na soap (1:1:1) on the surface of Teflon, aluminium and glass; **a–c** surfactant solutions in double-distilled water; **d**–**f** surfactant solution at pH 7. The* vertical line* indicates the CMC value of respective surfactant mixture

For mixtures with the best adsorption and micellization parameters, i.e., the solutions of AE:Na soap and AE:APG with molar ratios of 1:2 and 2:1 respectively, the contact angles were measured on three types of surfaces (Teflon, aluminium, glass). In addition, the wetting properties were also examined for the ternary mixtures, i.e., AE:APG:Na soap (molar ratio 1:1:1). The contact angle values obtained for the tested solutions are presented in Fig. 3a–c.

As can be noted, the highest wetting surface was the glass surface. On the all isotherms the maximum contact angle on the glass corresponds to the mixed surfactants concentration lower than CMC but close to that at which the saturated monolayer at the solution–air interface is formed. A similar behavior was previously observed on the glass surface for solutions of individual non-ionic surfactants [21]. The contact angles values on the three plates rapidly decrease after reaching the CMC. With increasing concentration, further gradual increase of the wettability was found. The two-component solutions in the highest range of concentration of surfactants have similar contact angle values (Fig. 3a, b). However, the mixture of AE:APG had the best wetting properties in a concentration range of 0.0004–0.001 mol/dm^3^, and the lowest values of contact angle on the surface of Teflon, aluminium and glass were as follows: 47.6°, 15.9° and 14.0° (Fig. 3a).

Studies carried out for the ternary mixture of AE:APG:Na soap indicate, that the best wetting properties can be observed in the concentration range of 0.0008–0.001 mol/dm^3^ (Fig. 3c). The concentrations were therefore higher as compared to the two-component mixtures. The lowest values of the contact angles on the surface of Teflon, aluminium and glass were as follows: 44.2°, 27.8° and 11.9°. It was stated that the solution with three surfactants in equimolar quantities improved wetting of the most hydrophobic (Teflon) and most hydrophilic (glass) surfaces, but the results were obtained for higher concentrations of the components.

Among the surfaces tested, Teflon was the least wetted surface. For wetting hydrophobic surfaces silicone surfactants (particularly trisiloxanes) are frequently used, which are called “superspreaders” [22]. However, the data indicate that the contact angle of silicone surfactants solutions on highly hydrophobic smooth Teflon AF coated silicon wafers is about 50–60°. Our research showed, that two- and three-component carbon based surfactant are also very effective in wetting hydrophobic Teflon surface.

### Effect of pH on the Surface Activity and Wetting Properties of the Surfactants Mixtures

**Fig. 4:**
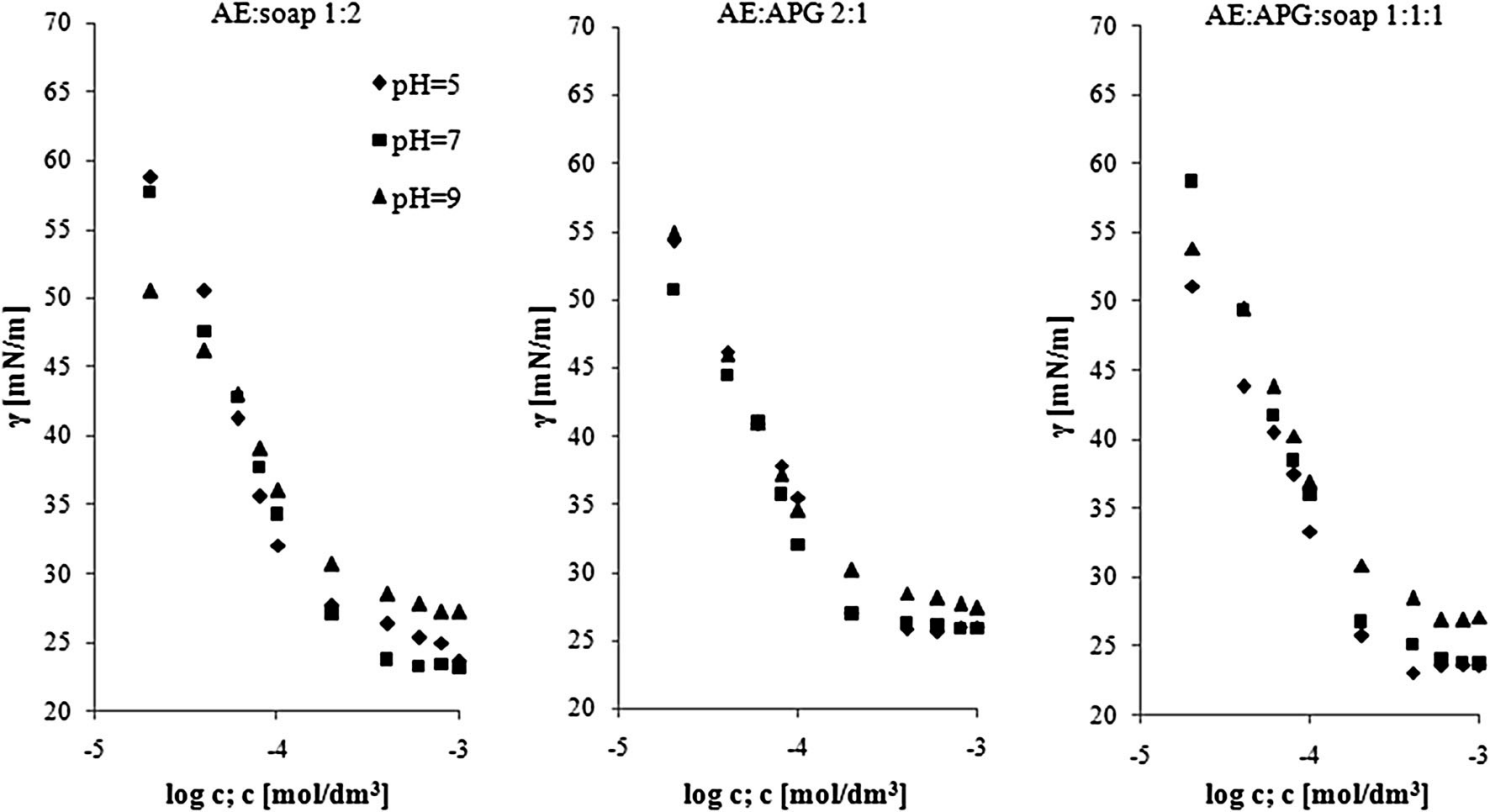
Surface tension *vs* the concentration of surfactants mixtures in solutions at pH 5, 7 and 9

In order to investigate the effect of pH on the surface activity of the surfactants mixtures, the binary and ternary solutions (i.e., AE:Na soap and AE:APG with molar ratios of 1:2 and 2:1, respectively and AE:APG:Na soap 1:1:1), were tested at different pH values. Figure 4 shows the results of the surface tension as a function of concentration of the surfactant mixture in buffers at pH 5, 7 and 9. All of the compositions reduced the surface tension below 30 mN/m in a very low range of concentrations (CMC approximately 1.5–3 × 10^−4^ mol/dm^3^) (Table 6). It indicates very good surface properties of the tested mixtures in acidic, basic and neural media.

**Table 6:** Selected parameters of surface activity and the intermolecular interactions in solutions of surfactant mixtures at different pH values

Surfactants (molar ratio)	pH	CMC × 10^4^ (mol/dm^3^)	γ_CMC_ (mN/m)	*p*C_20_	*X*_1_	$\beta_{LL}^{\sigma }$	$\ln \left( {C_{1}^{0} /C_{2}^{0} } \right)$	$X_{1}^{M}$	$\beta_{LL}^{M}$	$\ln \left( {{\text{CMC}}_{1}^{0} /{\text{CMC}}_{2}^{0} } \right)$
AE: soap (1:2)	5	1.23 ± 0.02	28.92 ± 0.11	4.5	0.5	−5.1	−1.1	0.6	−6.9	−1.9
	7	2.33 ± 0.07	23.96 ± 0.08	4.6	0.5	−4.1	−1.1	0.6	−4.2	−1.9
	9	2.41 ± 0.02	29.21 ± 0.40	4.8	0.5	−2.5	−1.1	0.6	−4.1	−1.9
AE:APG (2:1)	5	2.26 ± 0.03	25.64 ± 0.27	4.7	0.7	−4.2	−1.8	0.7	−3.2	−1.9
	7	1.77 ± 0.10	26.92 ± 0.29	4.8	0.7	−4.9	−1.8	0.7	−4.4	−1.9
	9	1.79 ± 0.06	29.53 ± 0.02	4.7	0.7	−3.8	−1.8	0.7	−4.4	−1.9
AE:APG:soap (1:1:1)	5	2.71 ± 0.15	23.03 ± 0.59	4.8	–	–	–	–	–	–
	7	2.04 ± 0.02	25.90 ± 0.39	4.5	–	–	–	–	–	–
	9	2.37 ± 0.16	28.97 ± 0.17	4.6	–	–	–	–	–	–

Among the AE:APG and three-component mixtures, the lowest CMC values were observed at pH 7. The solutions showed the lowest value of γ_CMC_ at pH 5. It was also found that all of the CMC values for AE:APG were above the CMC obtained in distilled water. The AE:soap mixtures showed reverse correlation: the lowest CMC at pH 5 and the lowest tension at neutral pH. From the data presented, it was found that the CMC for the AE:soap mixtures increased with increasing pH.

The results obtained for all tested mixtures of surfactants prepared in solutions at pH 5, 7 and 9 confirm the synergism in both: lowering the interfacial tension and creating mixed micelles (Table 6). Furthermore, mixtures of AE:APG molar ratio of 2:1 prepared in solutions at pH 5 and pH 7 demonstrated the effectiveness in lowering the interfacial tension.

The next stage of the work was to determine the wetting properties of the mixed surfactants at different pH values. The highest power of wetting was observed at neutral pH on three surfaces (Teflon, aluminium, glass). The results of the contact angle values for solutions at pH 7 are shown in Fig. 3d–f. As can be seen, the AE:soap solutions on the Teflon and aluminium showed a decrease in contact angle values with increasing concentration to approx. 0.0008 and 0.0006 mol/dm^3^ respectively (Fig. 3d). The mixture of AE:APG showed lower contact angle value on the Teflon plate even in lower concentration (0.0004 mol/dm^3^) (Fig. 3e). In the case of the glass surface, a gradual decrease in contact angle was measured, after exceeding concentration of approx. 0.0002 mol/dm^3^. The lowest contact angles values for the AE:soap mixture on the surface of Teflon, aluminium and glass were as follows: 48.6°, 6.3° and 4.4°. For the AE:APG solutions even lower values of Θ were obtained on the tested surfaces (44.1°, 3.5° and 4.0° respectively). For comparison, the lowest values of contact angles for the solutions made at pH = 5 and 9 did not exceed 50° and 12° on Teflon and metal/glass respectively (data not shown). Therefore a strong improvement in the wetting power was obtained at pH 7 as compared to solutions at other pH values and in distilled water. In addition, in our study Θ values measured on the hydrophilic plates are characterized by an almost perfectly wetting liquids (Θ close to zero).

The contact angle measurements were also made for ternary solutions deposited on the same surfaces. It can be concluded that this composition had a very similar wetting characteristic on the aluminium and glass surfaces (Fig. 3f). The lowest contact angles values on these plates were 5.5° and 8.0° respectively. These values are not lower than Θ obtained for the binary mixtures, but show also very good wetting properties of the AE:APG:soap mixture on the glass and aluminium. In contrast, three-component solutions at pH 7 had lower wetting power on Teflon compared to solutions with distilled water.
